# Clinical diagnostic utility of CA 15-3 for the diagnosis of malignant pleural effusion: A meta-analysis

**DOI:** 10.3892/etm.2014.2039

**Published:** 2014-10-30

**Authors:** QIANG WU, MIN LI, SHU ZHANG, LU CHEN, XINGTING GU, FENG XU

**Affiliations:** 1 Cancer Center, West China Hospital, Sichuan University, Chengdu, Sichuan 610041, P.R. China; 2 Department of Respiratory Medicine, West China Hospital, Sichuan University, Chengdu, Sichuan 610041, P.R. China

**Keywords:** CA 15-3, malignant pleural effusion, diagnosis, meta-analysis

## Abstract

Malignant pleural effusion (MPE) is one of the most common pleura-associated conditions observed in clinical practice. The development of MPE usually defines advanced cancer with a poor prognosis. Carbohydrate antigen 15-3 (CA 15-3), as an effective pleural fluid biomarker, has been an object of ongoing research in the detection of MPE. The aim of this meta-analysis was to establish the overall diagnostic accuracy of the measurement of pleural CA 15-3 for diagnosing MPE. The databases Medline (using PubMed as the search engine), Embase, Ovid, Web of Science and Cochrane database (up to December 2013) were searched to identify relevant studies. No lower date limit was applied. All literature published in English was reviewed. Sensitivity, specificity, likelihood ratio and diagnostic odds ratio (DOR) were pooled using a random-effect model. Summary receiver operating characteristic (SROC) curve analysis was conducted to evaluate the overall diagnostic value. The methodological quality was assessed in line with the Quality Assessment for Studies of Diagnostic Accuracy statement. Twenty-one studies with a total of 2,861 cases were included in present meta-analysis. The sensitivity, specificity, positive likelihood ratio (PLR), negative likelihood ratio (NLR) and DOR of CA 15-3 in the diagnosis of MPE were 0.58 [95% confidence interval (CI), 0.56–0.61], 0.91 (95% CI, 0.90–0.93), 8.93 (95% CI, 4.45–17.93), 0.46 (95% CI, 0.37–0.56) and 24.89 (95% CI, 10.39–59.63), respectively. In addition, the area under the curve (AUC) was 0.84. In conclusion, due to the significantly high specificity of pleural CA 15-3 in detecting MPE, it may play a pivotal role in screening to identify patients who may benefit from further invasive pathologic examination, particularly in those presenting clinical manifestations of MPE but with negative cytological findings of the pleural fluid. However, ruling out MPE by testing CA15-3 alone is not recommended due to its limited sensitivity, and it is recommended that the results of CA15-3 assays are interpreted in parallel with conventional test results and other clinical findings.

## Introduction

Pleural effusion is commonly found in patients suffering from various kinds of disease, and malignancy is one of the most frequent causes of pleural effusion (1). Pleural effusion may occur with almost all types of cancers, of which lung cancer is responsible for ~30% of cases (2). The etiological diagnosis and differential diagnosis between malignant pleural effusion (MPE) and benign pleural effusion is of significant importance due to the differences in treatment and prognosis between these two types of pleural effusions. Development of MPE usually defines advanced cancer with a poor prognosis, and moreover, current strategies for the diagnosis of MPE are clearly inadequate (3). As a standard method for the diagnosis of MPE, conventional pleural fluid cytology findings are positive in only 60% of cases on average (4). Additionally, closed pleural biopsy merely confers a small additive diagnostic value (5,6). Thoracoscopy has a high diagnostic accuracy and is able to establish the diagnosis in ~95% of cases; however, this interventional procedure may not be well accepted among patients and may not be available at all facilities (7).

Carbohydrate antigen 15-3 (CA 15-3), a glycoprotein expressed in normal and malignant tissues, including breast, lung and ovary cancer, has been found to be associated with malignancy and its secretion is increased in the presence of a tumor (8). It is easily detected in various biological specimens such as blood, serous liquid and tissue samples (9). Thus an increasing number of studies consider it to be a biomarker for diagnosing MPE (10–12). Although the accuracy of CA 15-3 in detecting MPE has been extensively studied, the precise diagnostic value remains unclear. Therefore, the present meta-analysis was performed to evaluate the overall accuracy of pleural CA 15-3 for diagnosing MPE.

## Materials and methods

### Identification of studies

The databases Medline (using PubMed as the search engine), Embase, Ovid, Web of Science and Cochrane database were searched to identify relevant studies published up to December 2013. No lower date limit was applied. Relevant references of articles were also searched manually. Relevant studies were identified using any of the following keywords: ‘tumor marker’, ‘carbohydrate antigen 15-3’, ‘CA 15-3’, ‘malignant pleural effusion’, ‘sensitivity’, ‘specificity’ and ‘accuracy’. The language of publication was limited to English. A study was included in the present meta-analysis if it provided both sensitivity and specificity data of CA 15-3 for the diagnosis of MPE. Letters to journal editors and conference abstracts, however, were excluded due to limited data. Two authors independently identified eligible studies when screening the searched studies. Any disagreements were arbitrated by a third author.

### Quality assessment

To assess the methodology of the included studies (13–33), the present meta-analysis was conducted in line with the Quality Assessment for Studies of Diagnostic Accuracy (QUADAS) statement (34). Two authors independently evaluated all included studies based on randomized sequence generation. Any disagreements were arbitrated by a third author.

### Data extraction

The data was extracted by two authors. The reviewers were blinded to publication details, such as author details, journal, patient characteristics, test method, cut-off value, sensitivity, specificity and methodological quality. Additionally, the following characteristics of study design for each article were retrieved: a) cross-sectional design (versus case-control design); b) consecutive or random sampling of patients; c) blinded (single or double) interpretation of determination and reference standard results; and d) prospective data collection. If no data on the above criteria were reported in the primary studies, these were treated as ‘Unknown’. Discrepancies were resolved by consensus. When multiple publications of the same study were identified, data was extracted as a single study.

### Statistical analysis

Standard methods recommended to provide diagnostic accuracy in meta-analyses were used (35). Then, the following indices of test accuracy were calculated for each study: sensitivity, specificity, positive likelihood ratio (PLR), negative likelihood ratio (NLR) and diagnostic odds ratio (DOR).

The sensitivity and specificity of each single test threshold identified for each study were used to plot a summary ROC (SROC) (36,37). Spearman’s rank correlation was performed as a test for threshold effect. The χ^2^ and Fisher’s exact tests were used to detect statistically significant heterogeneity across the studies. The average sensitivity, specificity and other related measurements of the studies were calculated using a random-effects model (38,39). To assess the effects of QUADAS score on the diagnostic ability of CA 15-3, it was included as a covariate in the meta-regression analysis (inverse-variance weighted). Effects of other covariates on DOR (cross-sectional design, consecutive or random sampling of patients, blind design, prospective data collection and assay method) were also analyzed. The relative DOR (RDOR) was calculated according to standard methods to analyze the change in diagnostic precision in the study per unit increase in the covariate (40,41). Publication bias was tested using funnel plots and the Deeks’ test (42). Statistical software packages were used to perform the analysis (Stata, version 8.2, Stata Corporation, College Station, TX, USA; Meta-Test version 0.6, New England Medical Center, Boston, MA, USA; and Meta-DiSc for Windows, XI Cochrane Colloquium, Barcelona, Spain).

## Results

### Description of studies

**Figure 1:**
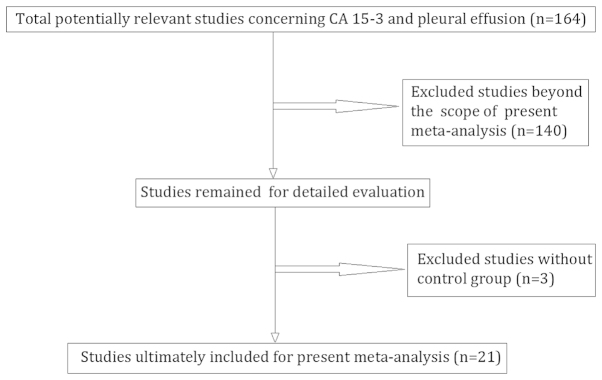
Flow chart of studies identified, included and excluded. CA 15-3, carbohydrate antigen 15-3.

Following the literature search, a total of 164 studies regarding CA 15-3 and pleural effusion were considered to be eligible for the present meta-analysis. Of these publications, 140 literature items that were beyond the scope of the present study were excluded and three articles without a control group were also excluded (43–45). Ultimately, 21 studies were selected with a total of 2,861 patients. A flow chart showing the selection of references for meta-analysis is shown in Fig. 1.

### Quality of included studies

In present meta-analysis, the average sample size of the included studies was 136 (range, 39 to 416). The diagnosis of MPE was made based on cytological or/and pathological findings, as the gold standard. Among the 21 studies, 15 (71.4%) had a cross-sectional design; in 16 studies (76.2%), the samples were collected from consecutive patients; 13 studies (61.9%) reported blinded interpretation of CA 15-3 assays independent of the reference standard; and 20 studies (95.2%) had a prospective study design. There were 13 studies with QUADAS scores ≥10 (Table I). As shown in Table I, P≥0.05 indicates values that did not reach statistical significance and these factors did not affect diagnostic accuracy. The clinical characteristics and other information are outlined in Tables II and III.

### Diagnostic accuracy

**Figure 2:**
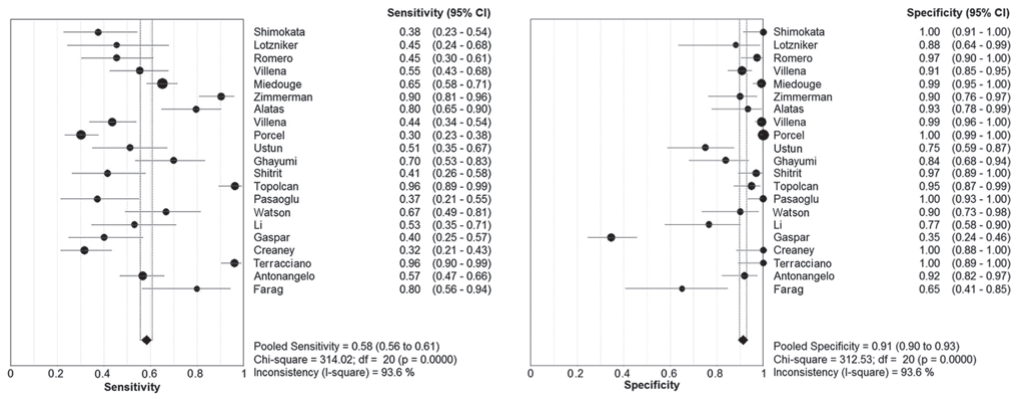
Forest plots of sensitivity and specificity for CA 15-3 assay for the diagnosis of MPE. The point estimates of sensitivity and specificity from each study are shown as solid circles. Error bars indicate 95% CI.

The forest plots of sensitivity and specificity of CA 15-3 assays in the various studies for the diagnosis of MPE are shown in Fig. 2. The sensitivity varied between 0.30 and 0.96 [pooled 0.58, 95% confidence interval (CI), 0.560–0.61], while the specificity ranged from 0.35 to 1.00 (pooled 0.91, 95% CI, 0.90–0.93). The PLR was 8.93 (95% CI, 4.45–17.93), the NLR was 0.46 (95% CI, 0.37–0.56) and the DOR was 24.89 (95% CI, 10.39–56.63). χ^2^ values of sensitivity, specificity, PLR, NLR and DOR were 314.02, 312.53, 201.71, 214.78 and 163.24, respectively, with all P-values <0.001, indicating significant heterogeneity between all studies.

**Figure 3:**
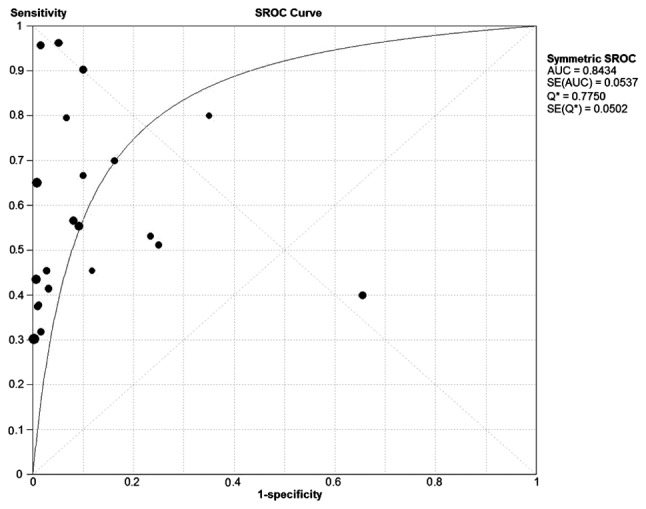
Summary receiver operating characteristic curve (SROC) of pleural CA 15-3 for the diagnosis of malignant pleural effusion. The size of each solid circle represents the sample size of each study. The regression SROC curve indicates the overall diagnostic accuracy. CA 15-3, carbohydrate antigen 15-3; AUC, area under the curve.

The Fig. 3 shows the SROC curve, which summarizes the test performance, and shows the balance between sensitivity and specificity. The Q-value is a global measure of test efficacy. It is the point of intersection of the SROC curve with a diagonal line from the left upper corner to the right lower corner of the ROC space, and corresponds to the highest common value of sensitivity and specificity for the test. This point does not indicate the only or even the best combination of sensitivity and specificity for a particular clinical setting, but represents an overall measure of the discriminatory power of a test. In present meta-analysis, the maximum joint sensitivity and specificity (the Q-value) was 0.77. The area under the curve (AUC) was 0.84.

**Figure 4:**
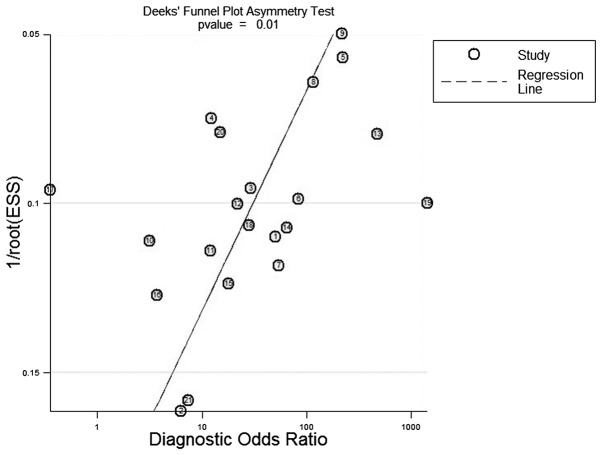
Funnel graph for the assessment of potential publication bias in studies concerning assays of CA 15-3 in pleural fluid for the diagnosis of malignant pleural effusion. CA 15-3, carbohydrate antigen 15-3; ESS, effective sample size.

When evaluating the publication bias, Deeks’ test was performed and it was significant (P=0.01). The funnel plot for publication bias (Fig. 4) is also essentially asymmetric. Thus, the results suggest a potential for publication bias.

## Discussion

MPE is one of the most common pleura-associated problems encountered in clinical practice. The diagnosis of malignancy in pleural effusions continues to be challenging (4). Finding an effectively diagnostic tool for MPE is of great importance. The detection of tumor markers such as CA 15-3 in the pleural fluid has been proposed as an alternative non-invasive method for the diagnosis of MPE (11).

The present meta-analysis investigated the overall diagnostic value of CA 15-3 measurements in pleural effusions in the diagnosis of MPE. The specificity was 0.91 (95% CI, 0.91–0.93), indicating a potential role for CA 15-3 analysis in the confirmation of MPE. In contrast with the high specificity, the sensitivity was only 0.58 (95% CI, 0.56–0.61), which is insufficient to exclude MPE. Thus, negative tests do not clearly indicate the absence of MPE, and patients with negative CA 15-3 results have a fairly high chance of having MPE. This trade-off has significant clinical implications.

To summarize the test performance, an SROC curve was generated, which indicates the balance between sensitivity and specificity (46). The SROC curve for the present meta-analysis revealed that the maximum joint sensitivity and specificity (Q value) was 0.77 and the AUC was 0.84, indicating that the overall accuracy was not very high.

The DOR, which is the ratio of the odds of a positive test result in the diseased relative to that in the non-diseased state, is an alternative indicator of test accuracy. The higher the DOR value, the more discriminatory the test (47). In the present meta-analysis, the mean DOR was found to be 24.89 (95% CI, 10.39–59.63), suggesting that CA 15-3 testing is a useful tool for aiding the diagnosis of MPE.

The PLR and NLR were also determined as measures of diagnostic accuracy in the present study, as likelihood ratios are considered to be more clinically meaningful than the DOR (48,49). A PLR value of 8.93 in the present study suggested that patients with MPE have an ~9-fold higher chance of testing positive in the CA 15-3 assay than patients without MPE, which should be helpful for in clinical practice. However, the NLR value of 0.46 determined in the present study meant that if the CA 15-3 assay result was negative, the probability that a patient was affected by MPE was ~46%, which is too high to rule out MPE.

In present study, QUADAS scores were used in the meta-regression to assess the effect of study quality on RDOR. As shown in Table I, the studies with higher quality (QUADAS ≥10) produced lower RDORs than those with lower quality. The meta-regression also indicated that differences between CA 15-3 assay methods (EIA versus non-EIA), studies with or without blinded design, cross-sectional, consecutive/random and prospective design, did not reach statistical significance. Thus, these factors do not affect diagnostic accuracy.

Notably, the results of the present study were consistent with those of a previous meta-analysis conducted by Liang *et al* (50). This earlier meta-analysis included 11 related studies prior to 2005. Since that time, additional studies on this field have been reported and are included in the present study. The search strategy and data extraction methods used in the present meta-analysis were more comprehensive than those in the previous meta-analysis, which was an important strength. Literature selection, quality assessment and data extraction were performed independently and in a blinded fashion by five reviewers. By meta-regression, the current study assessed the effects of covariates such as CA 15-3 assay methods on the RDOR, which was not performed in the previous meta-analysis (50). However, the present study had certain limitations. Firstly, studies published in languages other than English, unpublished studies and abstracts from conference proceedings were not included, which was probably a cause of publication bias. Secondly, issues such as the exact condition of the patients and laboratory infrastructure were not analyzed due to limited data.

The present meta-analysis suggested that the diagnostic value of CA 15-3 for MPE was far from perfection. Combining CA 15-3 with other markers may be an appropriate method for improving the diagnostic accuracy. The study by Romero *et al* found that the sensitivity and specificity of carcinoembryonic antigen combined with CA 15-3 in pleural fluid were 71% and 96%, respectively, which was better than testing CA 15-3 alone (15). Another study reported that the combination of thymidine kinase with CA 15-3 and procalcitonin appeared to be an optimal combination, nearly enabling differential diagnosing in all types of effusion (25).

In conclusion, with significantly high specificity in the detection of MPE, pleural CA 15-3 analysis is likely to be a effective assay to ascertain the potential existence of MPE in patients, particularly those who present clinical manifestation of MPE but with negative cytological findings of the pleural fluid, and thus provide the benefit of further invasive pathologic examination when elevated CA 15-3 levels are found in pleural effusions. However, ruling out MPE by testing CA15-3 alone is not recommended due to its limited sensitivity, and the results of CA15-3 assays should be interpreted in parallel with conventional test results and other clinical findings.

## Figures and Tables

**Table I:** Weighted meta-regression of the effects of methodological quality, study design and assay method on diagnostic precision of pleural CA 15-3 in 21 assays.

Covariates	No. of studies	Coefficient	RDOR (95% CI)	P-value
QUADAS ≥10	13	−1.788	0.17 (0.01–3.50)	0.226
Cross-sectional design	15	0.515	1.67 (0.21–13.22)	0.599
Consecutive or random	16	1.351	3.86 (0.22–66.57)	0.324
Blinded	13	2.353	10.51 (0.72–153.81)	0.080
Prospective	19	−1.154	0.32 (0.01–11.65)	0.502
Assay method (EIA/non-EIA)	7/14	0.198	1.22 (0.16–9.24)	0.836

CA 15-3, carbohydrate antigen 15-3; QUADAS, Quality Assessment for Studies of Diagnostic Accuracy; EIA, enzyme immunoassay; RDOR, relative diagnostic odds ratio; CI, confidence interval.

**Table II:** Summary of included studies.

Study/year (ref.)	Patient no.		Assay method	Cut-off	Test results				QUADAS score
	
	MPE	non-PE			TP	FP	FN	TN	
Shimokata/1988 (13)	40	41	RIA	16 U/ml	15	0	25	41	12
Lotzniker/1991 (14)	22	17	EIA	25 U/ml	10	2	12	15	7
Romero/1996 (15)	42	73	EIA	25 U/ml	20	2	24	71	12
Villena/1996 (16)	65	142	EIA	42 U/ml	36	1	75	129	12
Miédougé/1999 (17)	215	121	RIA	36.2 U/ml	140	1	75	120	9
Zimmerman/2000 (18)	72	40	EIA	Unknown	65	4	7	36	10
Alataş/2001 (19)	44	30	RIA	14 U/ml	35	2	9	28	13
Villena/2003 (20)	101	151	EIA	45 U/ml	44	1	57	150	12
Porcel/2004 (21)	166	250	RIA	75U/ml	50	0	116	250	12
Ustün/2004 (22)	41	40	EIA	30 U/ml	21	10	20	30	8
Ghayumi/2005 (23)	40	37	EIA	21.1 U/ml	28	6	12	31	12
Shitrit/2005 (24)	44	72	EIA	30 U/ml	17	2	24	62	11
Topolcan/2007 (25)	81	77	EIA	7.6 kIU/l	77	4	3	74	10
Paşaoğlu/2007 (26)	35	54	EIA	53 U/ml	13	0	22	54	9
Wagner/2007 (27)	36	30	ECIA	22.38 ng/ml	24	3	12	27	13
Li/2007 (28)	32	30	EIA	35 kU/l	17	7	15	23	10
Gaspar/2008 (29)	40	84	EIA	62.4 IU/l	16	55	24	29	11
Creaney/2008 (30)	76	30	EIA	53 kU/l	24	0	52	30	8
Terracciano/2010 (31)	103	32	EIA	41 kU/l	99	0	4	32	9
Antonangelo/2010 (32)	113	62	ECIA	27 kU/l	64	5	49	57	8
Farag/2012 (33)	20	20	ECIA	35 kU/l	16	7	4	13	7

MPE, malignant pleural effusion; RIA, radioimmunoassay; EIA, enzyme immunoassay; ECIA, electrochemiluminescence method; TP, true-positive; FP, false-positive; FN, false-negative; TN, true-negative; QUADAS, Quality Assessment for Studies of Diagnostic Accuracy.

**Table III:** Characteristics of included studies.

Study/year (ref.)	Country	Reference standard	Cross-sectional design	Consecutive and/or random?	Blinded design	Prospective
Shimokata/1988 (13)	Japan	Histology	Yes	Yes	Yes	Yes
Lotzniker/1991 (14)	Italy	Histology	Unknown	Unknown	Unknown	Unknown
Romero/1996 (15)	Spain	Histology	Unknown	Yes	Yes	Yes
Villena/1996 (16)	Spain	Histology	Unknown	Yes	Yes	Yes
Miédougé/1999 (17)	France	Histology	Yes	Yes	No	No
Zimmerman/2000 (18)	USA	Histology	Yes	Yes	Unknown	Yes
Alataş/2001 (19)	Turkey	Histology	No	Yes	Yes	Yes
Villena/2003 (20)	Spain	Histology	Unknown	Yes	Yes	Yes
Porcel/2004 (21)	Iran	Histology	Yes	Yes	Yes	Yes
Ustün/2004 (22)	Turkey	Histology	Unknown	No	Unknown	Yes
Ghayumi/2005 (23)	Iran	Histology	Yes	Yes	Yes	Yes
Shitrit/2005 (24)	Israel	Histology	Yes	Yes	Yes	Yes
Topolcan/2007 (25)	Czech	Histology	Yes	Yes	Yes	Yes
Paşaoğlu/2007 (26)	Turkey	Histology	Yes	Yes	Unknown	Yes
Wagner/2007 (27)	Brazil	Histology	Yes	Yes	Yes	Yes
Li/2007 (28)	China	Histology	Yes	Unknown	Yes	Yes
Gaspar/2008 (29)	Spain	Histology	Yes	Yes	Unknown	Yes
Creaney/2008 (30)	Australia	Histology	Yes	Yes	Yes	Yes
Terracciano/2010 (31)	Italy	Histology	Yes	Unknown	Yes	Yes
Antonangelo/2010 (32)	Brazil	Histology	Yes	Yes	Unknown	Yes
Farag/2012 (33)	Egypt	Histology	Yes	Unknown	No	Yes
